# Cold Stress-Induced Adipose Tissue Remodeling in *Min pigs*: Involvement of the *ADRB3*-ERK Axis, Autophagy, and Inflammatory Pathways

**DOI:** 10.3390/biom16101464

**Published:** 2026-10-08

**Authors:** Shuo Yang, Heshu Chen, Dongjie Zhang, Xinmiao He, Hong Ma, Zhenhua Guo, Liang Wang, Bo Fu, Wentao Wang, Di Liu

**Affiliations:** 1 Key Laboratory of Combining Farming and Animal Husbandry of Ministry of Agriculture and Rural Affairs, Heilongjiang Academy of Agricultural Sciences, Harbin 150086, China; 2 College of Animal Science and Technology, Northeast Agricultural University, Harbin 150030, China

**Keywords:** cold stress, *ADRB3*-ERK axis, autophagy, inflammation, adipose tissue beiging

## Abstract

Cold stress induces adaptive remodeling of adipose tissue, yet the coordinated molecular network, particularly the interplay between upstream signaling pathways and downstream cellular responses, remains incompletely understood. This study investigated whether cold stress regulates adipose tissue beiging, apoptosis, and systemic metabolic remodeling through autophagy and inflammation mediated by the β3-adrenergic receptor (*ADRB3*)-extracellular signal-regulated kinase (ERK) signaling axis. The results demonstrated that cold stress significantly increased energy expenditure and markedly improved glucose tolerance and insulin sensitivity in *Min pigs*. In subcutaneous adipose tissue, cold stress induced pronounced beiging, as evidenced by reduced adipocyte size, the presence of multilocular lipid droplets, and upregulation of beige adipocyte marker genes, accompanied by increased preadipocyte apoptosis. Transcriptomic analysis revealed significant enrichment of pathways related to autophagy and lipolysis, with inflammation (notably the TNF signaling pathway) also identified as a potential contributing process. Mechanistically, cold stress activated the *ADRB3*-ERK signaling axis, leading to enhanced autophagic activity, and inflammation-related genes were also upregulated, suggesting that inflammation may serve as a candidate process cooperating with autophagy. In conclusion, this study reveals a novel molecular mechanism whereby cold stress activates the *ADRB3*-ERK signaling axis to stimulate autophagy, with inflammation potentially cooperating as a candidate process, thereby driving adipose tissue beiging, apoptosis, and systemic metabolic remodeling.

## 1. Introduction

Adipose tissue is a critical metabolic organ that plays dual roles in energy storage and systemic metabolic regulation. Its remarkable functional plasticity, including beiging, lipolysis, and apoptosis, is crucial for enabling adaptation to environmental challenges [1,2]. Among these challenges, cold stress represents a prevalent environmental stressor in livestock production. Exposure to cold triggers profound metabolic reprogramming to maintain core body temperature, with adipose tissue remodeling serving as a pivotal adaptive response [3,4]. Accumulating evidence indicates that cold-induced adipose tissue beiging and apoptosis contribute to increased energy expenditure and metabolic activity [5], whereas dysregulation of these processes is closely associated with metabolic disorders such as insulin resistance and obesity [6]. Despite these advances, the molecular network coordinating adipose tissue beiging, apoptosis, and metabolic remodeling under cold stress remains incompletely elucidated, particularly the interplay among upstream signaling receptors, kinase cascades, and downstream cellular pathways.

The β3-adrenergic receptor (*ADRB3*), a G protein-coupled receptor predominantly expressed in adipose tissue, is widely recognized as a core receptor for cold-induced metabolic adaptation [7]. Activation of *ADRB3* by cold stress or selective agonists has been shown to promote lipolysis, mitochondrial biogenesis, and beige adipocyte formation [8]. Previous studies have demonstrated that *ADRB3* signaling can induce cellular senescence through cAMP-dependent protein kinase A (PKA) activation [9], and that *ADRB3*-deficiency impairs cold tolerance and energy expenditure [10]. However, beyond the PKA-dependent pathway, whether *ADRB3* engages additional kinase cascades to regulate adipose tissue remodeling under cold stress remains unclear. The extracellular signal-regulated kinase (ERK) pathway, a conserved mitogen-activated protein kinase (MAPK) cascade, plays a critical role in regulating cell proliferation, apoptosis, and metabolic processes [11,12]. Emerging evidence suggests that ERK activation participates in cold-induced autophagy and inflammation [13,14], but the link between *ADRB3* and ERK signaling and their coordinated role in regulating adipose tissue beiging and apoptosis under cold stress have not been systematically investigated.

Despite these advances, whether *ADRB3* engages ERK signaling to coordinate autophagy and inflammation during cold stress remains unknown. Autophagy and inflammation are two tightly interconnected cellular processes regulating adipose tissue homeostasis [15,16]. Cold stress has been shown to induce autophagy in adipose tissue, a process necessary for beige adipocyte maturation and energy metabolism [17,18]. One study demonstrated that autophagy facilitates lipid droplet degradation and mitochondrial quality control during the beiging process [19], while inhibition of autophagy attenuates cold-induced energy expenditure [20]. Concurrently, cold stress elicits a mild inflammatory response in adipose tissue, which may act as a signaling hub promoting metabolic adaptation [21,22]. For instance, TNF-α signaling, a key inflammatory pathway, has been reported to enhance lipolysis and adipose tissue beiging by activating downstream kinase cascades [23]. Despite these findings, the molecular mechanisms underlying the co-activation of autophagy and inflammation under cold stress, and how they synergistically regulate adipose tissue beiging and apoptosis, remain poorly understood. Notably, whether the *ADRB3*-ERK axis serves as an upstream regulator of autophagy and inflammation in cold-stressed adipose tissue represents a critical gap in current research.

The *Min pig*, a Chinese indigenous breed renowned for its exceptional cold tolerance, provides an ideal animal model for investigating adipose tissue adaptation to cold stress [24,25]. Previous studies have reported that *Min pigs* exhibit enhanced metabolic flexibility and improved insulin sensitivity in response to cold exposure [26], but the underlying cellular and molecular mechanisms remain largely undefined. In the present study, we hypothesized that cold stress triggers adipose tissue beiging, apoptosis, and metabolic remodeling through *ADRB3*-ERK axis-mediated activation of autophagy and inflammation. To test this hypothesis, we systematically examined the effects of cold stress on systemic metabolism, adipose tissue morphology, and molecular signaling in *Min pigs* and further validated the regulatory roles of *ADRB3*, ERK, autophagy, and inflammation using an in vitro preadipocyte model.

Herein, we report that cold stress enhances systemic metabolism and insulin sensitivity, induces adipose tissue beiging and preadipocyte apoptosis, and activates autophagic and inflammatory pathways in *Min pigs*. Mechanistically, our findings reveal that *ADRB3* signaling activates the ERK cascade, which in turn mediates autophagy and inflammation to drive adipose tissue remodeling. Collectively, this study provides novel insights into the molecular network governing cold-induced adipose tissue adaptation and identifies the *ADRB3*-ERK-autophagy/inflammation axis as a potential therapeutic target for improving metabolic health and cold tolerance in both livestock and humans.

## 2. Materials and Methods

### 2.1. Experimental Animals

Thirty-day-old weaned male Min piglets were obtained from the breeding farm of the Heilongjiang Academy of Agricultural Sciences. Piglets were randomly assigned to either a room-temperature control group (RT; 25 °C, *n* = 5) or a cold stress treatment group (CS; 4 °C, *n* = 5). The cold stress treatment lasted for 3 consecutive days. Prior to experimental procedures, all animals were individually housed in metabolic cages for 24 h.

### 2.2. Hormone Level Measurement

Blood samples were collected from the anterior vena cava of piglets from different treatment groups following restraint. Serum was separated after coagulation. Hormone levels were determined using an Insulin ELISA Kit (ml002341, mlbio, Shanghai, China), an Irisin ELISA Kit (ml037372, mlbio, Shanghai, China), and a Leptin ELISA Kit (ml002355, mlbio, Shanghai, China) according to the manufacturer’s instructions.

### 2.3. Glucose Tolerance and Insulin Sensitivity Tests

Glucose Tolerance Test (GTT): After fasting for 5 h, piglets were intravenously injected via the auricular vein with a glucose solution at a dose of 0.5 g/kg body weight. Blood samples were collected at 0, 15, 30, 60, and 120 min post-injection, and blood glucose concentrations were measured using a glucometer.

Insulin Tolerance Test (ITT): After fasting for 5 h, piglets were intravenously injected via the auricular vein with an insulin solution at dose of 0.75 U/kg body weight. Blood samples were collected at 0, 15, 30, 60, and 120 min post-injection, and blood glucose concentrations were measured using a glucometer.

### 2.4. Indirect Calorimetry and Energy Expenditure Measurement

Piglets were individually housed in metabolic cages equipped with water and feed troughs, with ad libitum access to water and food. Whole-body oxygen consumption was measured using an open-circuit indirect calorimetry system with automatic temperature and light control, and data were recorded for 72 h.

### 2.5. Detection of Cell Apoptosis Rate by Flow Cytometry

Transfected preadipocytes were digested with trypsin without ethylenediaminetetraacetic acid (EDTA). The cell apoptosis rate was detected using an Annexin V-FITC/PI Kit (HY-K1073, MCE, Shanghai, China) according to the manufacturer’s instructions. After being washed with pre-cooled phosphate-buffered saline (PBS), cells were resuspended in 500 μL of apoptosis-inducing buffer and incubated. For different treatment groups, 10 μL of PI and 5 μL of Annexin V/Alexa Fluor 488 were added, respectively, followed by incubation at room temperature in the dark. Voltage was adjusted using a blank control first, then compensation was set, and finally, the treatment groups were analyzed.

### 2.6. Morphological Observation of Adipose Tissue and TUNEL Assay for Apoptosis Detection

Subcutaneous adipose tissue samples were collected from piglets and cut into approximately 0.5 cm^3^ cubes, followed by fixation in 4% paraformaldehyde solution overnight. The samples were subsequently dehydrated through a graded ethanol series, with each step lasting 30 min and cleared in xylene. Tissues were then infiltrated with low-melting-point paraffin followed by high-melting-point paraffin and then embedded in paraffin blocks. Paraffin sections were prepared, stained with Hematoxylin and Eosin (H&E), and finally mounted. For TUNEL staining, paraffin sections were prepared, stained with TUNEL, and finally mounted. Images were captured using a microscope (DP40; OLYMPUS, Tokyo, Japan).

### 2.7. Overexpression of ADRB3 and siRNA Knockdown of ADRB3

The full-length coding sequence of porcine *ADRB3* (NM_001099927.1) was amplified using porcine cDNA and primers (Supplementary Table S1). Subsequently, the purified PCR products were inserted between the HindIII and EcoRI sites of the pcDNA3.1(+) vector (Promega, Madison, WI, USA) to construct pcDNA3.1(+)-*ADRB3*. Following construction, the plasmids were confirmed by DNA sequencing. The stromal vascular fraction (SVF) cells were then transfected with 1.25 µg of pcDNA3.1(+)-*ADRB3* or pcDNA3.1(+) empty vector, followed by RT-qPCR analysis to assess gene expression.

For *ADRB3* knockdown, SVF cells were transfected with commercially available siRNAs targeting *ADRB3* (GENERALBIOL, Wuhan, China) using Lipofectamine 2000 (11668-019, Thermo Fisher Scientific, Waltham, MA, USA) at a final concentration of 100 nM. Cells were harvested 24 h post-transfection for gene expression analysis to validate *ADRB3* knockdown.

### 2.8. Isolation and Culture of Preadipocytes

Subcutaneous adipose tissue was collected from 30-day-old piglets under sterile conditions. The tissue was carefully minced and digested with 2.5 mg/mL collagenase I in DMEM/F12 medium (PM150312, Pricella, Wuhan, Hubei, China) at 37 °C for 1–2 h. The digest was filtered through a 50 μm cell strainer, washed twice with plain DMEM/F12, and finally cultured in DMEM/F12 supplemented with 10% fetal bovine serum (209111, NEST, Wuxi, Jiangsu, China), 100 U/mL penicillin, and 100 μg/mL streptomycin in a standard cell culture incubator with 5% CO_2_. Cells were passaged upon reaching 90% confluency and passages 3 to 4 were used for subsequent experiments. Adipogenic differentiation was induced by treating confluent preadipocytes with differentiation medium (DMEM/F12 supplemented with 10% FBS, 0.5 mM 3-isobutyl-1-methylxanthine, 1 μM dexamethasone, 10 μg/mL insulin, and 1 μM rosiglitazone) for 2 days, followed by maintenance in medium containing 10 μg/mL insulin for an additional 6 days, with the medium changed every 2 days.

### 2.9. Real-Time Quantitative PCR (RT-qPCR)

Total RNA was isolated from preadipocytes using Trizol reagent (420810, Ambion, Beijing, China) according to the manufacturer’s instructions. RNA was then quantified and reverse-transcribed into cDNA using a PrimeScript™ RT Reagent Kit (RR047a, Takara, Beijing, China). RT-qPCR was performed using the Ultra SYBR One Step RT-qPCR Kit (01170, Cwbio, Beijing, China). Relative mRNA expression levels were calculated using the 2^−ΔΔCt^ method and normalized to the internal control GAPDH.

### 2.10. Western Blot Analysis

Western blot analysis was performed to assess the protein expression levels of LC3-I, LC3-II, ERK1/2, phosphorylated ERK1/2 (p-ERK1/2), *ADRB3*, PGC1α, and UCP3. Subcutaneous adipose tissue samples from the RT and CS groups were lysed in 200 μL of radioimmunoprecipitation assay (RIPA) buffer (R0010, Solarbio, Beijing, China). Protein concentrations were quantified using a BCA assay kit (KTD3001, Abbkine, Wuhan, Hubei, China). Subsequently, equal amounts of protein were separated by 10% SDS-PAGE gels and transferred onto PVDF membranes (IPVH00010, Millipore, Carrigtwohill, County Cork, Ireland). Membranes were blocked with 5% non-fat milk and incubated with primary antibodies against ERK1/2 (1:1000, A4782, Abclonal, Wuhan, Hubei, China), p-ERK1/2 (1:2000, AP0485, Abclonal, Wuhan, Hubei, China), LC3-I/LC3-II (1:1000, ER1907-46, HUABIO, Hangzhou, China), *ADRB3* (1:1000, ab94506, Abcam, Shanghai, China), PGC1α (1:1500, ab191838, Abcam, Shanghai, China), UCP3 (1:1000, ab180643, Abcam, Shanghai, China), and β-actin (1:2000, ab8227, Abcam, Shanghai, China). After incubation with horseradish peroxidase-conjugated goat anti-rabbit immunoglobulin G (AB125938, Abcam, Beijing, China) at room temperature for 1 h, protein bands were visualized using an enhanced chemiluminescence (ECL) kit (HY-K2006, MCE, Shanghai, China). Band intensities were quantified using ImageJ software (version 1.52a), with β-Tubulin (100109-MCR05, Sino Biological, Beijing, China) serving as the loading control.

### 2.11. Fluorescence Microscopy

Cells were seeded, cultured, and differentiated in 6-well plates. To visualize lipid droplets, live cells were washed with warm medium and incubated with AdipoRed™ Assay Reagent (PT-7009, Lonza, Basel, Switzerland) for 15 min, followed by fixation in 4% paraformaldehyde. Subsequently, an antifade mounting medium with DAPI was added to the slides, and images were captured using a microscope (DP40; OLYMPUS, Tokyo, Japan).

### 2.12. mRNA Sequencing, RNA-Seq Data Analysis, and Functional Analysis

Library preparation and RNA-seq were performed by Biomarker Technologies Co., Ltd. (Beijing, China). cDNA libraries were constructed using RNA samples with high purity (OD260/280 ≥ 2.0) and integrity (RIN > 7). Differentially expressed genes (DEGs) were identified based on a fold change (FC) > 1.5 and *p* < 0.05 following multiple testing correction. Gene set enrichment analysis (GSEA) was performed to identify enriched pathways in each comparison group. Statistical analysis and data visualization were conducted using an online platform (https://cloud.oebiotech.cn/task/, accessed on 23 October 2025).

### 2.13. Data Analysis

All experiments were conducted with a minimum of three biological replicates. Statistical analysis was performed using GraphPad Prism software (version 6.01). Differences between groups were evaluated using Student’s *t*-test or one-way analysis of variance (ANOVA). Statistical significance was defined as * *p* < 0.05, ** *p* < 0.01, *** *p* < 0.001, and **** *p* < 0.0001.

### 2.14. Use of Generative AI Tools

During the preparation of this manuscript, the authors used DeepSeek-V3 (DeepSeek AI, version V3) for English-language polishing of the full manuscript. The tool was used solely to improve grammar, spelling, punctuation, clarity, and readability.

## 3. Results

### 3.1. Cold Stress Enhances Systemic Metabolism and Insulin Sensitivity in Min pigs

**Figure 1:**
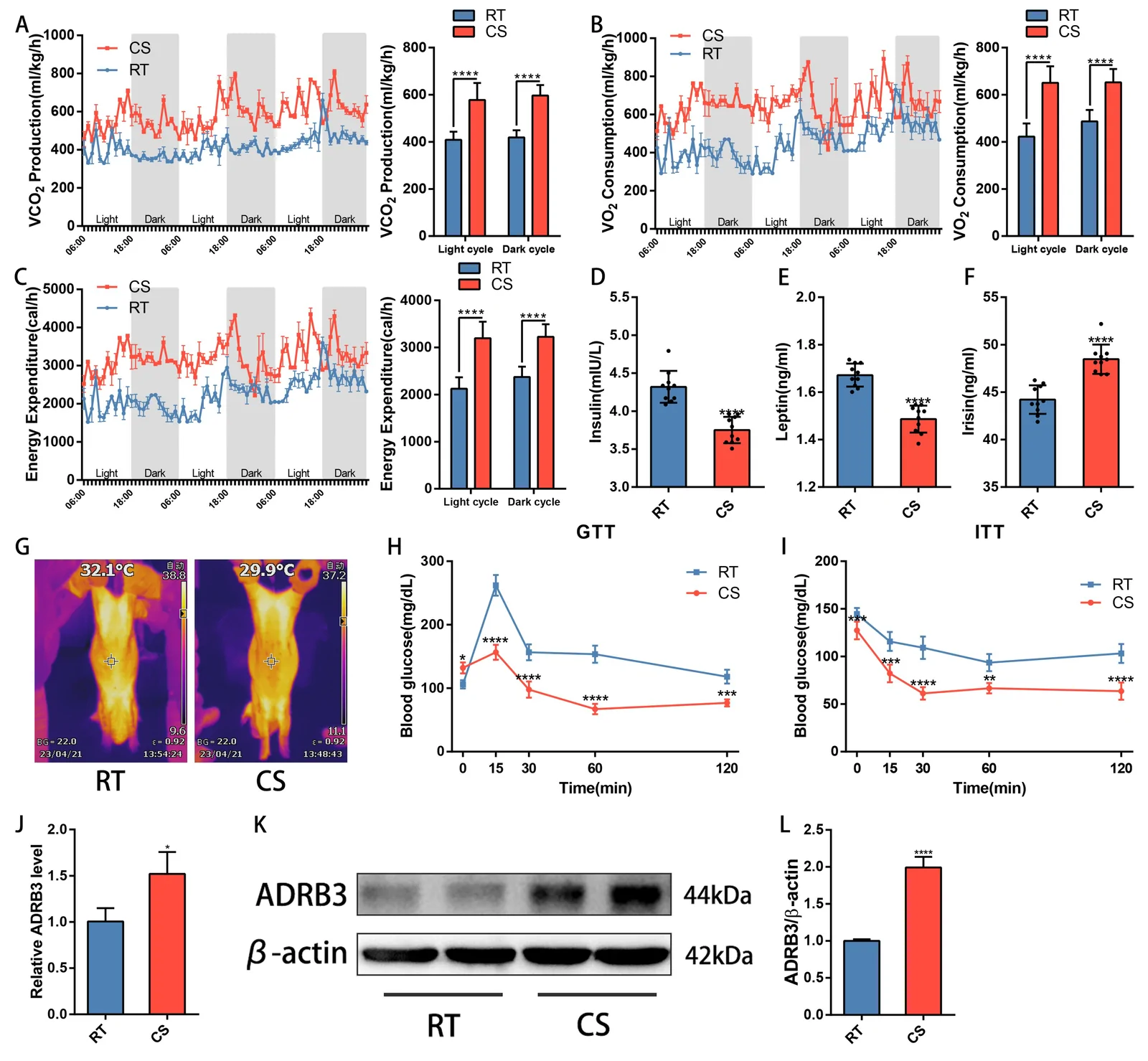
The effect of cold stress on metabolic homeostasis in *Min pigs*. (**A**) CO_2_ production volume; (**B**) O_2_ consumption volume; (**C**) energy expenditure; (**D**) insulin level; (**E**) leptin level; (**F**) irisin level; (**G**) infrared thermography. The Chinese term “自动” at the top right means “automatic temperature scale”; (**H**) glucose tolerance test (GTT); (**I**) insulin sensitivity. (**J**) mRNA expression level of *ADRB3*. (**K**) Protein expression level of *ADRB3*. (**L**) Quantitative analysis of grayscale values. (* *p* < 0.05, ** *p* < 0.01, *** *p* < 0.001, **** *p* < 0.0001). The original western blot images can be found in Supplementary materials.

Cold stress significantly enhanced systemic metabolic activity in Min piglets. Compared with the RT group, the CS group showed sustained increases in CO_2_ production, O_2_ consumption, and energy expenditure (*p* < 0.001), with average increases of 40.4%, 42.0%, and 42.8%, respectively (Figure 1A–C). Notably, no pronounced circadian rhythm differences were observed in either group for these parameters (Figure 1A–C), suggesting a weak regulatory effect of the diurnal cycle on the basal metabolism of piglets. Furthermore, infrared thermographic analysis revealed a significantly higher body surface temperature in the CS group compared with the RT group (29.9 °C vs. 32.1 °C; Figure 1G), confirming an enhanced metabolic response to cold stress.

Cold stress altered the secretion pattern of serum metabolism-related hormones in Min piglets. Serum insulin and leptin concentrations were markedly reduced in the CS group compared to the RT group (*p* < 0.001; Figure 1D,E), whereas irisin levels were significantly elevated (*p* < 0.001; Figure 1F).

Cold stress also markedly enhanced glucose tolerance and insulin sensitivity in Min piglets. In the GTT, blood glucose concentration prior to glucose administration was significantly higher in the CS group than in the RT group (*p* < 0.05), suggesting that cold stress promotes endogenous glucose production. Following glucose injection, blood glucose concentration was significantly lower in the CS group than in the RT group (*p* < 0.01; Figure 1H), indicating an enhanced capacity for glucose disposal. Consistently, results from the ITT demonstrated a more pronounced reduction in blood glucose concentration after insulin administration in the CS group than in the RT group (*p* < 0.01; Figure 1I), confirming that cold stress significantly improves insulin sensitivity in piglets. In the CS group, *ADRB3* mRNA expression in adipose tissue was increased compared with the RT group (Figure 1J). Consistently, *ADRB3* protein expression in adipose tissue was also elevated (Figure 1K,L).

### 3.2. Cold Stress and β3-Adrenergic Stimulation Induce Apoptosis in Adipose Tissue and Preadipocytes

**Figure 2:**
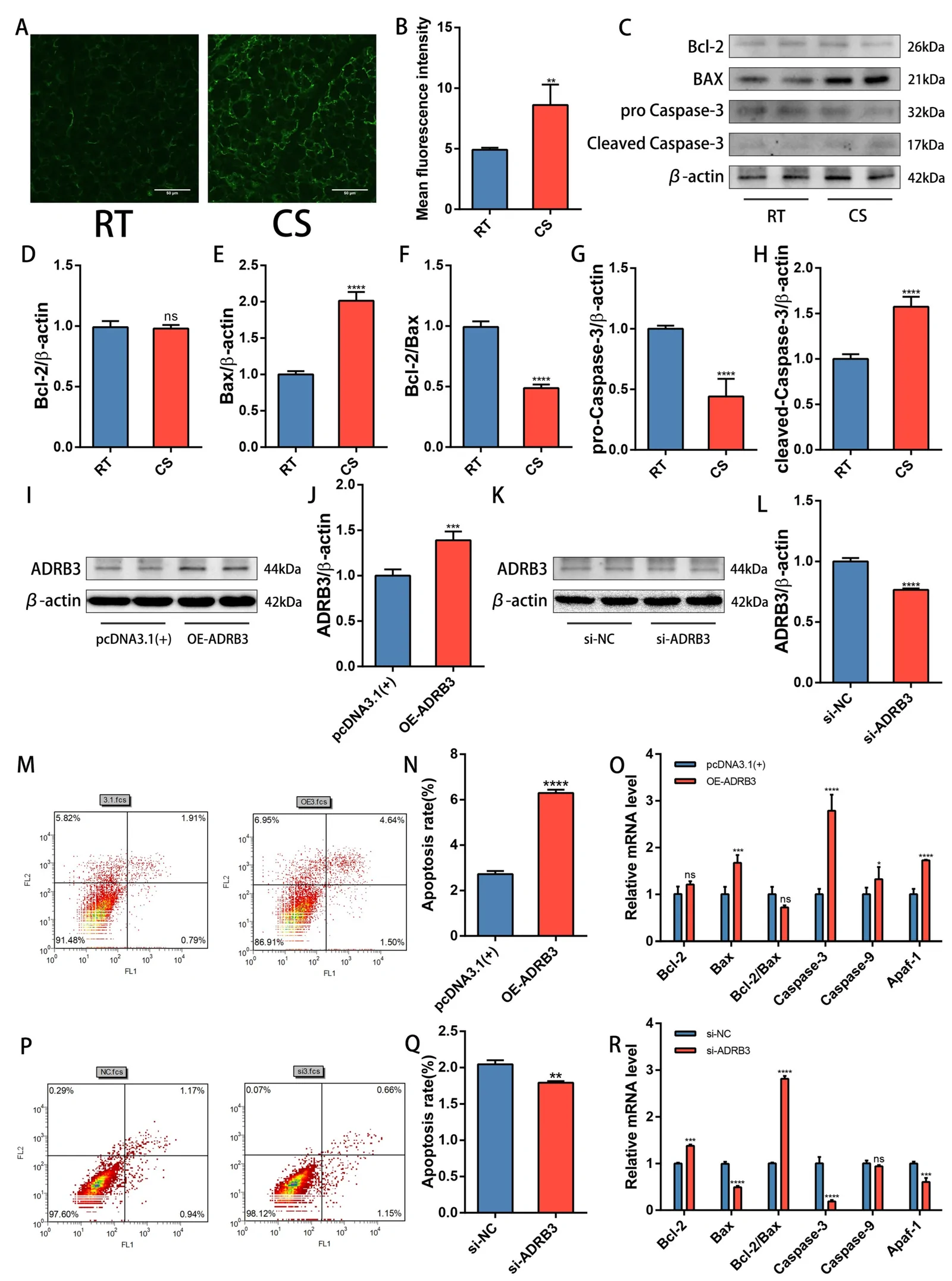
Effects of cold stress on adipose tissue apoptosis in vivo and *ADRB3*-simulated cold stress on preadipocyte apoptosis in vitro. (**A**,**B**) Representative TUNEL fluorescence images and corresponding quantitative analysis of fluorescence intensity in the RT and CS groups. (**C**–**H**) Western blots and quantitative analysis of apoptosis-related protein expression in the RT and CS groups. (*n* = 4–6). (**I**,**J**) Western blot analysis of *ADRB3* level after overexpression of *ADRB3*. (**K**,**L**) Western blot analysis of *ADRB3* level after knockdown of *ADRB3*. (*n* = 4–6). (**M**) Representative flow cytometry plots of cells transfected with an empty vector or an *ADRB3*-overexpressing vector. (**N**) Quantitative analysis of apoptosis rates by flow cytometry. (**O**) Expression levels of apoptosis-related genes following transfection with an empty vector or an *ADRB3*-overexpressing vector. (*n* = 4–6). (**P**) Representative flow cytometry plots of cells treated with NC or *ADRB3*-knockdown reagents. (**Q**) Quantitative analysis of apoptosis rates by flow cytometry. (**R**) Expression levels of apoptosis-related genes following treatment with NC or *ADRB3*-knockdown reagents (n = 4–6). (* *p* < 0.05, ** *p* < 0.01, *** *p* < 0.001, **** *p* < 0.0001).

To determine whether cold stress induces apoptosis in adipose tissue, TUNEL staining was performed on adipose tissue dissected from piglets housed under cold stress (CS) and room temperature (RT). The CS group showed significantly enhanced TUNEL fluorescence intensity compared with the RT group (Figure 2A,B), indicating increased apoptosis. We further examined the protein levels of apoptosis-related genes in adipose tissue. Western blot analysis revealed no obvious change in *Bcl-2* protein levels, whereas *Bax* protein levels were increased, resulting in a decreased *Bcl-2/Bax* ratio. In addition, pro-*caspase-3* levels were decreased and cleaved *caspase-3* levels were increased in the CS group (Figure 2C–H). These results indicate that cold stress promotes apoptosis in adipose tissue.

To confirm the efficiency of *ADRB3* modulation, *ADRB3* protein levels were measured in preadipocytes after *ADRB3* overexpression and knockdown. Overexpression of *ADRB3* significantly increased *ADRB3* protein levels, whereas *ADRB3* knockdown markedly decreased *ADRB3* protein levels (Figure 2I–L).

To elucidate the regulatory effect of *ADRB3* on preadipocyte apoptosis under cold stress-mimicking conditions, apoptosis was first assessed by flow cytometry after *ADRB3* overexpression (OE-*ADRB3*). Compared with the empty vector control group (pcDNA3.1(+)), the apoptosis rate was significantly higher in the OE-*ADRB3* group (*p* < 0.01; Figure 2M,N), suggesting a pro-apoptotic effect of *ADRB3*. RT-qPCR analysis further revealed that the *Bcl-2/Bax* ratio was less than 1 in the OE-*ADRB3* group, accompanied by marked upregulation of the pro-apoptotic genes *Caspase-3* and *Apaf-1* (*p* < 0.001; Figure 2O). Collectively, these results indicate that *ADRB3* overexpression effectively promotes apoptosis in preadipocytes.

To verify the functional specificity of *ADRB3*, apoptosis was further assessed by flow cytometry after *ADRB3* knockdown (si-*ADRB3*). Compared with the negative control group (si-NC), preadipocytes in the si-*ADRB3* group exhibited a significantly lower apoptosis rate (*p* < 0.01; Figure 2P,Q). Consistently, RT-qPCR analysis of apoptosis-related genes revealed significant upregulation of the anti-apoptotic gene *Bcl-2* (*p* < 0.01) and downregulation of the pro-apoptotic gene Bax, resulting in a markedly elevated Bcl-2/Bax ratio (*p* < 0.01). In addition, the relative expression of the pro-apoptotic gene Caspase-3 was significantly reduced (*p* < 0.05; Figure 2R). Collectively, the flow cytometric and gene expression results demonstrate that *ADRB3* knockdown significantly inhibits preadipocyte apoptosis.

### 3.3. Cold Stress Promotes the Beiging of Adipose Tissue

**Figure 3:**
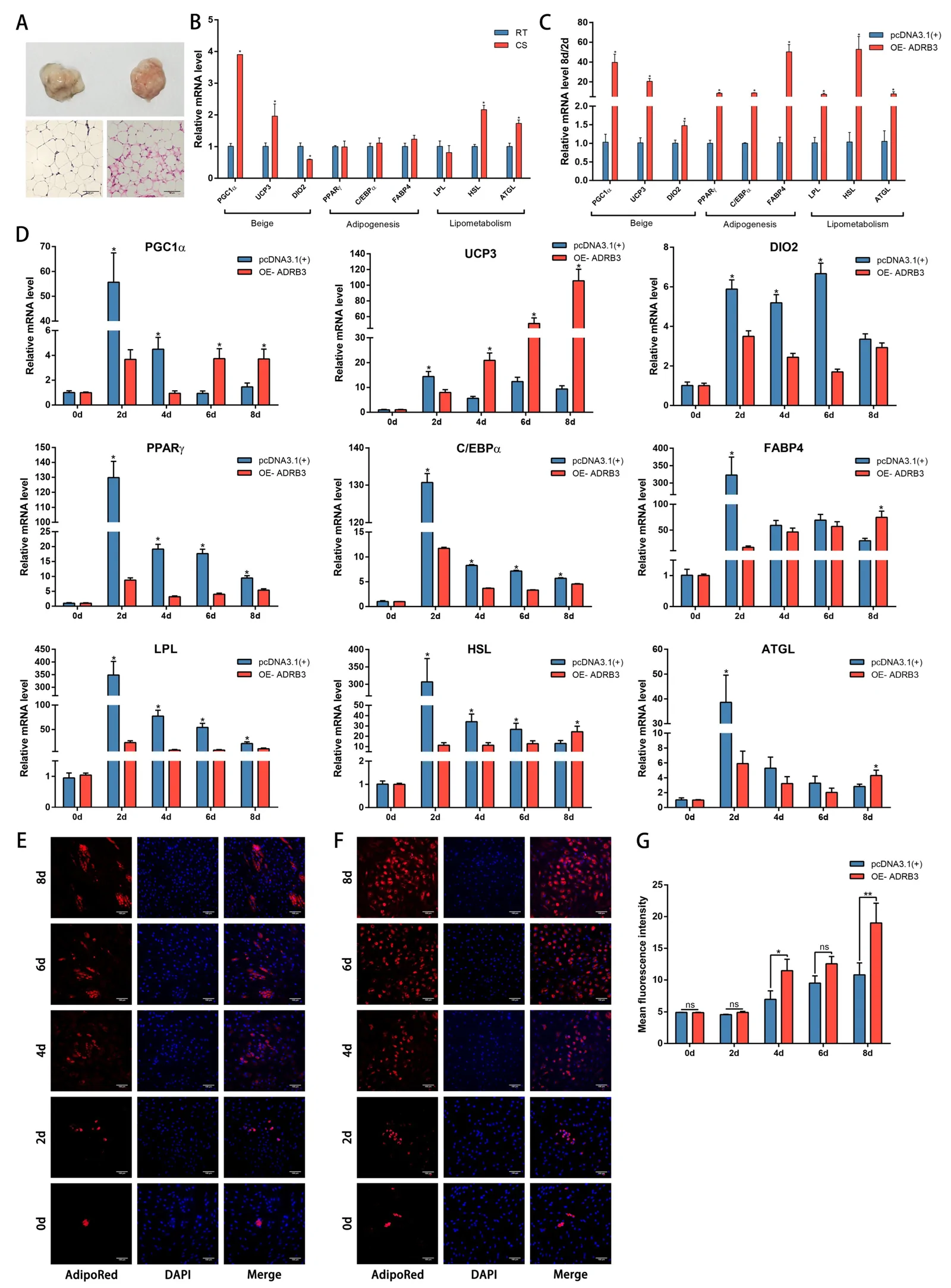
Cold stress induces adipose tissue beiging both in vivo and in vitro. (**A**) Representative macroscopic images and H&E-stained sections of subcutaneous adipose tissue. Scale bar = 50 μm. (**B**) mRNA expression levels of beige adipocyte marker genes, adipogenic genes, and lipid metabolism-related genes in adipose tissue under cold stress. (**C**) Relative mRNA expression (day 8 vs. day 2) of beige adipocyte marker genes, adipogenic genes, and lipid metabolism-related genes during differentiation in preadipocytes transfected with an empty vector or an *ADRB3*-overexpressing vector (*n* = 4–6). (**D**) Temporal mRNA expression profiles (days 0, 2, 4, 6, and 8) of beige adipocyte marker genes, adipogenic genes, and lipid metabolism-related genes during differentiation induction in preadipocytes transfected with an empty vector or an *ADRB3*-overexpressing vector (*n* = 4–6). (**E**) Lipid droplet morphology and DAPI-stained nuclei in preadipocytes of the pcDNA3.1(+) group at days 0, 2, 4, 6, and 8. Scale bar = 100 μm. (**F**) Lipid droplet morphology and DAPI-stained nuclei in preadipocytes of the *ADRB3*-overexpression group at days 0, 2, 4, 6, and 8. Scale bar = 100 μm. (**G**) Relative fluorescence intensity of (**E**,**F**). (* *p* < 0.05, ** *p* < 0.01).

To investigate the effect of cold stress on adipose tissue beiging, analyses were conducted at both the tissue and cellular levels. Under cold stimulation, the subcutaneous adipose tissue from *Min pigs* exhibited a visibly darker appearance. H&E staining revealed a more compact tissue structure characterized by reduced adipocyte size, increased numbers of small lipid droplets and a higher proportion of multilocular adipocytes (Figure 3A).

The mRNA expression levels of genes related to adipose tissue beiging, adipogenesis and lipid metabolism were quantified in adipose tissues from the CS and RT groups. Compared with the RT group, the CS group exhibited a significantly higher expression of the beige adipocyte marker genes *UCP3* and *PGC1α* (*p* < 0.01), whereas the expression of *DIO2* was significantly decreased (*p* < 0.01). No significant differences were observed in the expression of adipogenesis-related genes, including *PPARγ*, *C/EBPα*, and *FABP4* (*p* > 0.01). Similarly, the lipid metabolism gene LPL showed no significant difference between groups (*p* > 0.01), whereas the expression of *HSL* and *ATGL* was significantly higher in the CS group than in the RT group (*p* < 0.01) (Figure 3B).

To mimic cold stimulation at the cellular level, *ADRB3* was overexpressed in preadipocytes, and relative gene expression levels were measured by RT-qPCR at day 8 (mature differentiation stage) and day 2 (initiated differentiation stage). The temporal differences in gene expression were quantified using the day 8/day 2 (8d/2d) expression ratio. The 8d/2d ratios of the beige adipocyte marker genes *PGC1α*, *UCP3*, and *DIO2* were significantly increased in the OE-*ADRB3* group (*p* < 0.01), indicating cold stress involvement in energy metabolism via mitochondrial thermogenesis. In addition, OE-*ADRB3* significantly elevated the expression levels of core adipogenic transcription factors, including *PPARγ* and *C/EBPα* (*p* < 0.01), as well as the mature adipocyte marker gene *FABP4* (*p* < 0.01). The expression pattern of lipid metabolism genes directly reflects the lipid-handling capacity of adipocytes. The lipid uptake gene *LPL* and the lipolysis genes *HSL* and *ATGL* were all significantly increased in the OE-*ADRB3* group (*p* < 0.01) (Figure 3C). Notably, while *DIO2* expression was significantly decreased in cold-stressed adipose tissue in vivo (Figure 3B), its 8d/2d ratio was increased upon *ADRB3* overexpression in vitro (Figure 3C). This discrepancy may reflect the complexity of the in vivo microenvironment, including potential compensatory mechanisms or indirect effects of other cell types within adipose tissue, warranting further investigation.

Detailed temporal analyses revealed distinct expression dynamics of beiging, adipogenesis, and lipid metabolism-related genes between the pcDNA3.1(+) and OE-*ADRB3* groups. In the pcDNA3.1(+) group, *PGC1α* expression peaked at day 2 and subsequently declined, whereas in the OE-*ADRB3* group, *PGC1α* expression gradually increased after day 2 and reached significantly higher levels at day 8 (*p* < 0.05); *UCP3* expression was significantly higher in the OE-*ADRB3* group at day 8 compared to the pcDNA3.1(+) group (*p* < 0.05); *DIO2* exhibited high expression at days 2 and 8 in the pcDNA3.1(+) group but showed lower expression at intermediate time points (days 4–6) in the OE-*ADRB3* group, indicating time-dependent expression patterns for beiging-related genes. For adipogenic markers, *PPARγ* expression was high at day 2 in the pcDNA3.1(+) group but upregulated after day 4 in the OE-*ADRB3* group (*p* < 0.05); *C/EBPα* was highly expressed at day 2 in the pcDNA3.1(+) group but remained consistently lower throughout differentiation in the OE-*ADRB3* group, suggesting distinct molecular regulation of adipogenesis under the two conditions. Distinct temporal patterns were also observed for lipid metabolism-related genes. *LPL* was elevated at day 2 in the pcDNA3.1(+) group but decreased at later stages (days 6–8) in OE-*ADRB3* group; *FABP4* was significantly increased at day 8 in OE-*ADRB3* group (*p* < 0.05); *HSL* reached its peak expression at day 8 in the OE-*ADRB3* group (*p* < 0.05); *ATGL* was high at day 2 in the pcDNA3.1(+) group but markedly reduced at later stages (days 6–8) in the OE-*ADRB3* group. Collectively, these results reveal spatiotemporal differences in the expression of lipid metabolism-related genes between the pcDNA3.1(+) and OE-*ADRB3* groups, reflecting the molecular basis for their distinct functional differentiation (Figure 3D).

The effect of the cold stress mimetic *ADRB3* on lipid droplet formation was investigated. Preadipocytes were transfected with either the empty vector pcDNA3.1(+) or the pcDNA3.1(+)-*ADRB3* plasmid. Lipid droplet morphology was observed at days 0, 2, 4, 6, and 8 of differentiation using the AdipoRed method (Figure 3E,F), and relative fluorescence intensity was quantified (Figure 3G). In both the pcDNA3.1(+) and OE-*ADRB3* groups, lipid droplet accumulation increased progressively over time. Notably, compared with the pcDNA3.1(+) group, cells in the OE-*ADRB3* group exhibited larger lipid droplet areas and a denser lipid droplet morphology, suggesting the generation of beige-like adipocytes. Quantification of relative fluorescence intensity revealed no significant difference between the two groups at days 0 and 2; however, at days 4, 6, and 8, relative fluorescence intensity was significantly higher in the OE-*ADRB3* group than in the pcDNA3.1(+) group, indicating the formation of more beige adipocytes.

### 3.4. Transcriptomic Profiling Reveals Cold Stress Regulates Autophagy, Inflammation, and Lipolysis Pathways

**Figure 4:**
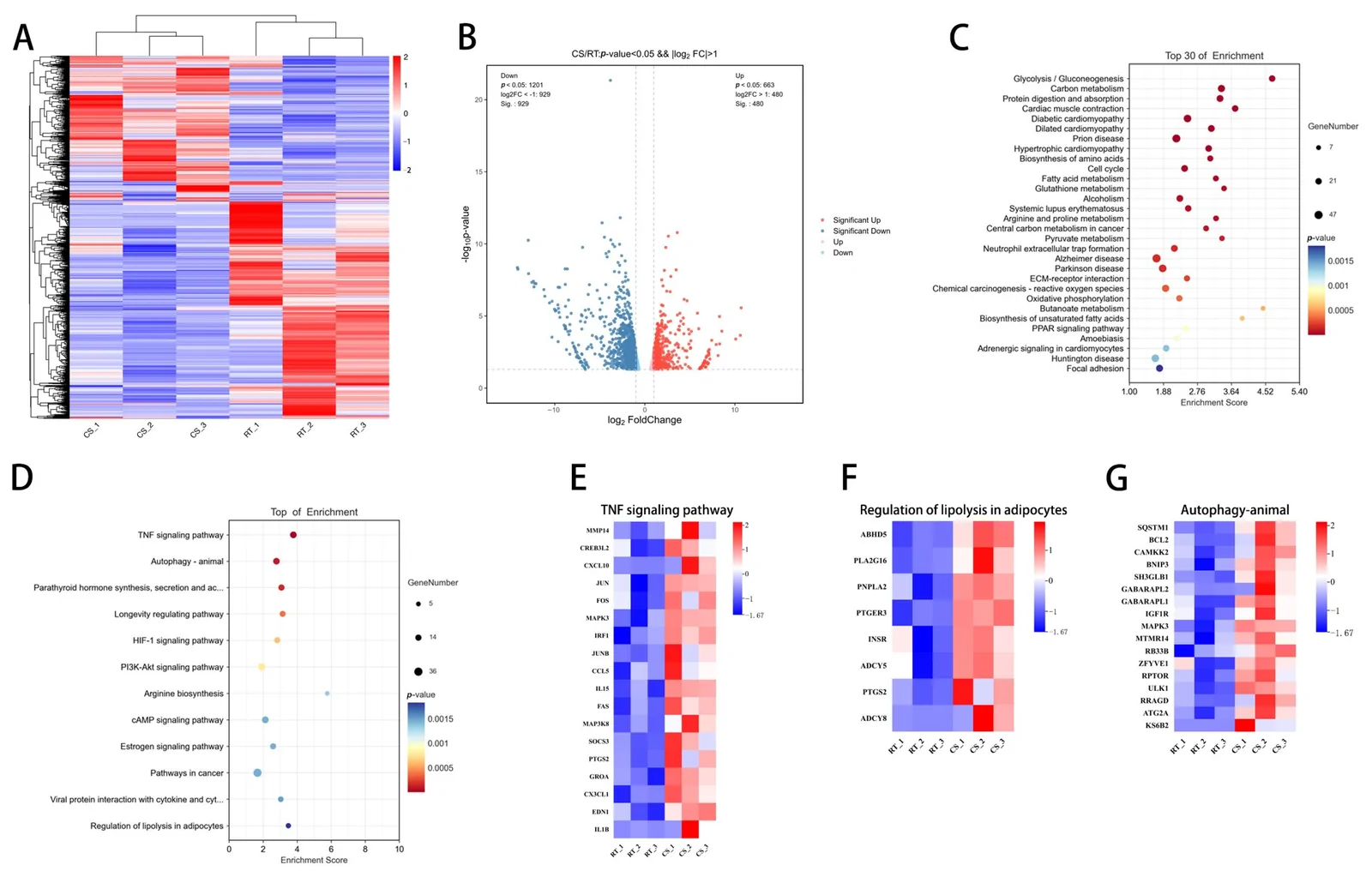
Transcriptomic analysis of adipose tissue changes under cold stress. (**A**) Clustering heatmap of gene expression profiles comparing RT and CS groups. (**B**) Volcano plot depicting differentially expressed genes (DEGs) between RT and CS groups. (**C**,**D**) KEGG pathway enrichment analysis of downregulated (**C**) and upregulated (**D**) genes in the CS group compared to the RT group. Dot plots display the most significantly enriched pathways, where dot color represents the adjusted *p*-value (Q-value) and dot size corresponds to the number of enriched DEGs. (**E**–**G**) Heatmaps showing expression patterns of genes within the enriched KEGG pathways: TNF signaling pathway (**E**), regulation of lipolysis in adipocytes (**F**), and autophagy in animals (**G**).

Cluster analysis of gene expression profiles from RT and CS adipose tissue showed a clear separation between the two groups (Figure 4A). Red and blue represent up- and downregulated genes, respectively. High intra-group consistency and pronounced inter-group differences indicate that cold stress induces extensive transcriptional remodeling of adipose tissue. The volcano plot (Figure 4B) comparing CS and RT samples identified a large number of DEGs, including both significantly upregulated (red points) and downregulated (blue points) transcripts (|log_2_FC| > 1, *p* < 0.05), whereas grey points represent non-significant genes. These results demonstrate that cold stress induces a broad transcriptional response involving extensive gene regulation. Downregulated genes in the CS group were enriched in pathways such as Glycolysis/Gluconeogenesis, Protein Digestion and Absorption, and Fatty Acid Metabolism, indicating suppression of adipose tissue glucose metabolism, protein-related functions, and fatty acid metabolism under cold exposure (Figure 4C). Upregulated genes in the CS group were significantly enriched in TNF signaling pathway, Autophagy (Animal), and Lipolysis pathways, suggesting activation of inflammatory signaling, autophagy, and lipolysis regulation as adaptive responses to cold stress (Figure 4D). Correspondingly, heatmaps were generated for genes in the TNF signaling pathway (Figure 4E), Lipolysis pathway (Figure 4F), and Autophagy pathway (Figure 4G), collectively indicating CS activates TNF-mediated inflammation, lipolysis, and autophagy pathways (as enriched in Figure 4D), thereby driving functional remodeling of adipose tissue.

### 3.5. GSEA and Mechanistic Validation Indicate Involvement of Inflammatory Pathway and ERK-Mediated Autophagy in Cold Stress Response

**Figure 5:**
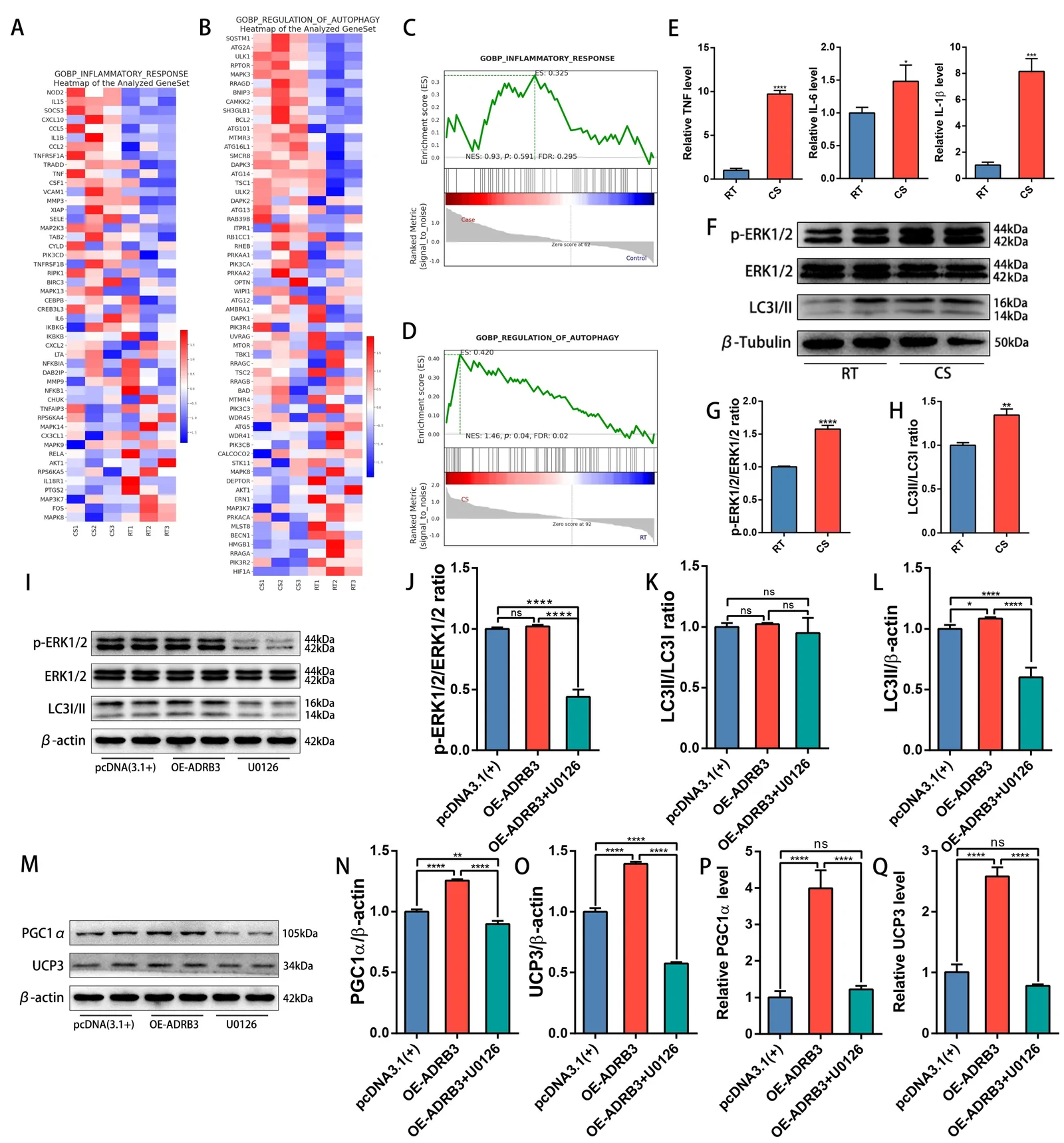
Involvement of inflammatory pathways and ERK-mediated autophagy in the cold stress response. (**A**–**D**) GSEA of RNA-seq data. (**A**,**C**) The inflammatory response gene set showed a non-significant enrichment trend in the CS group (NES = 0.93, *p* = 0.591, FDR = 0.295). (**B**,**D**) The autophagy gene set was significantly enriched in the CS group (NES = 1.46, *p* = 0.04, FDR = 0.02). (**E**) mRNA expression levels of inflammatory response genes (*n* = 4–6). (**F**–**H**) Representative Western blots and densitometric analysis of ERK1/2, p-ERK1/2, and LC3I/II protein levels in the RT and CS groups (*n* = 4–6). (**I**–**L**) Representative Western blots and densitometric analysis of ERK1/2, p-ERK1/2, and LC3-I/II protein levels in the pcDNA3.1(+) control, *ADRB3*-overexpressing, and U0126-treated groups (*n* = 4–6). (**M**–**O**) Representative Western blots and densitometric analysis of PGC1α and UCP3 protein levels in the pcDNA3.1(+) control, *ADRB3*-overexpressing, and U0126-treated groups (*n* = 4–6). (**P**,**Q**) mRNA expression level of PGC1α and UCP3(*n* = 4–6). (* *p* < 0.05, ** *p* < 0.01, *** *p* < 0.001, **** *p* < 0.0001).

This section analyzes the expression patterns of gene sets related to inflammatory response and autophagy in adipose tissue under cold stress. Gene set enrichment analysis (GSEA) of the inflammatory response gene set in CS and RT groups is presented in Figure 5A. The inflammatory response gene set did not show statistically significant enrichment in the CS group (NES = 0.93, *p* = 0.591, FDR = 0.295). However, the enrichment score (ES) was 0.325, with the peak appearing on the left side, suggesting that core genes tended to be highly expressed in the CS group, which may indicate a tendency toward inflammatory pathway activation in response to cold exposure (Figure 5C). To further assess inflammatory activation, the mRNA levels of the inflammatory markers *TNF*, *IL-6*, and *IL-1β* were measured and were all elevated in the CS group compared with the RT group (Figure 5E). The heatmap for the autophagy-related gene set is shown in Figure 5B. Autophagy-related genes exhibited significant enrichment in the CS group (Figure 5D), with an NES of 1.46 (*p* = 0.04, FDR = 0.02), suggesting that cold stress significantly upregulated the expression of autophagy-related genes in adipose tissue, indicative of the activation of the autophagy pathway under cold stress. To further explore upstream signaling mechanisms regulating autophagy, the ERK1/2 pathway was examined. The ratio of p-ERK1/2 to total ERK1/2 (Figure 5G) was significantly higher in the CS group compared to the RT group (*p* < 0.001), indicating the activation of the ERK1/2 signaling pathway under cold stress. Analysis of the autophagy marker *LC3* demonstrated that the *LC3-II/I* ratio (Figure 5H) was significantly higher in the CS group than in the RT group (*p* < 0.01), reflecting enhanced autophagic activity (Figure 5F–H). Collectively, these results demonstrate that cold stress markedly induces autophagy-related gene expression and autophagic activity in adipose tissue, at least in part through activation of the ERK1/2 signaling pathway, while inflammatory signaling also shows a tendency toward activation under cold stress.

At the cellular level, we investigated changes in the ERK and autophagy pathways after *ADRB3* overexpression and treatment with the ERK inhibitor U0126 (Figure 5I–L). *ADRB3* overexpression alone did not alter the p-ERK1/2/ERK1/2 ratio, whereas U0126 treatment decreased this ratio, confirming that U0126 inhibited ERK signaling. The LC3-II/I ratio showed no significant change following *ADRB3* overexpression or U0126 treatment; however, the LC3-II/β-actin ratio was elevated, indicating that inhibition of the ERK pathway suppresses autophagy.

We further examined the effects of *ADRB3* overexpression and U0126 treatment on beige adipocyte marker genes (Figure 5M–Q). *ADRB3* overexpression increased both mRNA and protein levels of PGC1α and UCP3, whereas inhibition of the ERK pathway with U0126 reduced their expression, indicating that the ERK pathway regulates beige adipocyte marker expression.

## 4. Discussion

This study systematically elucidated the molecular mechanisms underlying cold-induced adipose tissue remodeling using a cold-tolerant indigenous *Min pig* model in vivo and a complementary preadipocyte in vitro. The central finding is that cold stress promotes adipose tissue beiging, preadipocyte apoptosis, and systemic metabolic remodeling through activation of the *ADRB3*-ERK axis, which potentially coordinates autophagy and inflammatory responses, with inflammation considered a candidate cooperating process. These findings reveal an integrated signaling network governing cold adaptation in adipose tissue and highlight the remarkable metabolic plasticity of *Min pigs*, and may provide mechanistic insights that could inform future research relevant to livestock production and metabolic disease; however, therapeutic applications were not evaluated in this study.

Cold stress is well recognized as a potent inducer of metabolic reprogramming to sustain thermogenesis, with adipose tissue acting as a central effector [3,4]. Consistent with previous studies in mice and humans [26,27], our results show that cold-exposed *Min pigs* exhibited significantly increased O_2_ consumption, CO_2_ production, and energy expenditure (Figure 1A–C), accompanied by enhanced glucose tolerance and insulin sensitivity (Figure 1H–I). Notably, the lack of circadian rhythm in metabolic parameters (Figure 1A–C) suggests cold stress alters the circadian regulation of basal metabolism in Min piglets. This alteration may represent an adaptive strategy to ensure a continuous energy supply for thermogenesis, an observation that has not previously been reported in cold-tolerant livestock breeds. Moreover, changes in the secretion of metabolic hormones, including downregulated insulin and leptin, and upregulated irisin (Figure 1D–F) further support the occurrence of metabolic adaptation. Irisin, a myokine/adipokine linked to beige adipocyte formation and enhanced insulin sensitivity [28], was significantly elevated in cold-stressed *Min pigs*, consistent with the induction of adipose tissue beiging (Figure 3). In contrast to rodent models where cold stress often induces transient insulin resistance [29], *Min pigs* exhibited improved insulin sensitivity, likely due to their inherent cold tolerance and efficient adipose tissue remodeling. This finding aligns with recent studies on Tibetan pigs [30], emphasizing that cold-adapted breeds have evolved distinct metabolic strategies to cope with low-temperature environments and represent valuable models for studying adaptive metabolism.

*ADRB3* is a well-characterized mediator of cold-induced thermogenesis [31,32], but its role in adipocyte apoptosis has remained controversial. The present study (Figure 2) clearly shows that *ADRB3* overexpression induces preadipocyte apoptosis by downregulating the *Bcl-2/Bax* ratio and upregulating the expression of *Caspase-3* and *Apaf-1*, while *ADRB3* knockdown exerts opposing effects. These findings align with findings by Ju et al. [33], who reported that cold-induced adipocyte apoptosis promotes metabolic adaptation, but extend previous work by identifying *ADRB3* as a direct upstream regulator of this process. While earlier studies primarily focused on *ADRB3*-mediated lipolysis and beiging via PKA signaling [32,34], our results revealed a previously underappreciated role for *ADRB3* in apoptosis regulation. The pro-apoptotic effect of *ADRB3* may facilitate adipose tissue remodeling by eliminating dysfunctional preadipocytes and creating a microenvironment conducive to beige adipocyte formation [33]. This dual role of *ADRB3* in regulating both apoptosis and beiging underscores its central function in cold-induced adipose tissue adaptation and represents the first systematic demonstration of this mechanism in a livestock species. However, a limitation is that receptor overexpression alone may not fully mimic physiological *ADRB3* activation by cold-induced sympathetic signaling, and pharmacological adrenergic stimulation, which activates downstream cAMP independently of *ADRB3*, was not tested; future comparisons of these treatments are needed to determine whether the apoptotic effects are specifically *ADRB3*-dependent or reflect downstream cAMP signaling.

Beiging of white adipose tissue constitutes a critical adaptive response to cold exposure [2,35]. Our results show that in *Min pigs*, cold stress induced pronounced morphological changes in adipose tissue, including reduced lipid droplet size and the emergence of multilocular adipocytes (Figure 3A), accompanied by increased expression of upregulated key beige adipocyte markers, such as *UCP3* and *PGC1α* (Figure 3B), consistent with the established findings in mice [36,37]. Notably, *ADRB3* overexpression mimicked cold-induced beiging (Figure 3C–F), confirming *ADRB3* as a key mediator of beige adipocyte formation in pigs. It should be noted that Figure 3C and Figure 3D use different normalizations and therefore answer different biological questions. In Figure 3D, the expression of all analyzed genes was markedly increased at day 2 relative to day 0, particularly in the pcDNA3.1(+) control group. We speculate that this is because day 0 cells were uninduced preadipocytes, whereas day 2 cells had already been exposed to differentiation induction for 2 days; consequently, day 2 expression increased sharply relative to day 0. OE-*ADRB3* may partially attenuate this culture condition-induced surge. Thus, using day 0 (uninduced preadipocytes) as the sole reference may not be fully comprehensive. To more comprehensively evaluate gene expression under induced differentiation conditions, we therefore plotted the day 8/day 2 ratios for each gene (Figure 3C), which better reflect temporal expression changes during preadipocyte adipogenic differentiation and reveal whether OE-*ADRB3* alters the differentiation trajectory rather than simply the absolute day 8 level. In contrast, Figure 3D shows the actual time-resolved expression levels at each differentiation stage. Therefore, an increased normalized day 8/day 2 ratio in Figure 3C does not necessarily indicate higher absolute expression at day 8; rather, it indicates an altered temporal induction pattern. This distinction explains the apparent discrepancy between Figure 3C and Figure 3D, in which the day 8 levels of *PPARγ*, *C/EBPα*, *DIO2*, and *LPL* were lower in OE-*ADRB3* cells than in control cells, whereas *FABP4* and *HSL* were higher and *PGC1α* and *UCP3* showed stage-specific increases. Spatiotemporal gene expression analysis (Figure 3D) further revealed that *ADRB3* regulates beiging-related genes in a time-dependent manner: *PGC1α* and *UCP3* were upregulated at later stages of differentiation (day 8), suggesting that *ADRB3* primarily promotes beige adipocyte maturation rather than initiation. Furthermore, *ADRB3* altered the temporal induction of lipolysis genes (*HSL*, *ATGL*) and the lipid uptake gene *LPL* (Figure 3C), and at day 8, *HSL* expression was higher whereas *LPL* expression was lower in OE-*ADRB3* cells than in control cells (Figure 3D), indicating gene-specific and coordinated regulation of lipid metabolism and beiging. This finding aligns with the work of Mottillo et al. [38], who reported that lipolysis provides fatty acids for mitochondrial thermogenesis in beige adipocytes, and further extended this concept by linking this process to *ADRB3* signaling in a livestock context. Notably, while *DIO2* expression was significantly decreased in cold-stressed adipose tissue in vivo, its 8d/2d ratio was increased upon *ADRB3* overexpression in vitro. This discrepancy may reflect the contribution of non-adipocyte cell types or systemic factors such as thyroid hormone availability in vivo, which are absent in the in vitro system. Future studies using co-culture or in vivo knockdown approaches are needed to resolve this complexity.

Transcriptomic analysis revealed that autophagy and lipolysis were among the most prominently upregulated pathways in cold-stressed adipose tissue, whereas inflammation-related signaling, notably TNF-α, was identified as a potential contributing process (Figure 4), consistent with previous reports that cold stress may induce mild inflammation and autophagy to facilitate metabolic adaptation [20,39]. GSEA further confirmed significant enrichment of autophagy-related genes (NES = 1.46, FDR = 0.02), while core inflammatory genes exhibited increased expression (Figure 5A,C), suggesting a potential cooperative involvement of these two processes. Mechanistically, cold stress activates ERK1/2 signaling (Figure 5F) and increases autophagic activity (Figure 5G), linking ERK to autophagy regulation. This finding aligns with that of Latorre-Muro et al. [40], who reported that ERK-dependent autophagy promotes beige adipogenesis, and further extended this model by identifying *ADRB3* as the upstream receptor initiating this cascade. Notably, the *ADRB3*-ERK axis appears to integrate autophagy and potentially inflammatory signaling, which may cooperatively contribute to adipose tissue beiging, apoptosis, and metabolic remodeling, which has not previously been described in the cold stress response. Given the well-documented reciprocal regulation between inflammation and autophagy in adipose tissue [15,16], our findings suggest that cold-induced mild inflammation may synergize with autophagy to enhance metabolic flexibility: autophagy facilitates lipid droplet degradation and mitochondrial quality control during beiging [39], while inflammation may activate downstream kinases that promote lipolysis and apoptosis [41]. Collectively, the *ADRB3*-ERK axis serves as a central hub potentially integrating these processes, thereby providing insights into how upstream adrenergic signaling coordinates downstream cellular responses under cold stress.

Cold stress poses a major challenge to livestock production by impairing feed efficiency, growth performance, and disease susceptibility [42]. The present findings suggest that modulation of the *ADRB3*-ERK axis may represent a promising strategy to enhance cold tolerance in livestock: selective breeding for elevated *ADRB3* expression or enhanced ERK activity might potentially improve metabolic adaptation to cold environments, thereby mitigating cold stress-induced economic losses by promoting adipose tissue remodeling and energy efficiency. Obesity and insulin resistance remain major global health concerns, and stimulation of beige adipogenesis has emerged as a potential therapeutic approach [43,44]. The *ADRB3*-ERK-autophagy/inflammation axis identified in this study offers a novel mechanistic framework for targeting metabolic diseases: *ADRB3* agonists could be optimized to activate ERK-dependent autophagy/inflammation, thereby promoting adipose tissue beiging and insulin sensitivity; while concomitant modulation of excessive inflammation without impairing autophagy activity may help maintain metabolic adaptation and tissue homeostasis.

## 5. Conclusions

This study demonstrates that cold stress triggers adipose tissue beiging, preadipocyte apoptosis, and systemic metabolic remodeling in *Min pigs* via the *ADRB3*-ERK axis-mediated activation of autophagy, with inflammation potentially participating as a cooperating process. These findings uncover a novel molecular network regulating cold adaptation and underscore the unique metabolic plasticity of cold-tolerant breeds. The *ADRB3*-ERK-autophagy/inflammation axis not only advances our understanding of adipose tissue biology but also provides practical targets for improving cold tolerance in livestock and developing therapeutic strategies for human metabolic disorders.

## Data Availability

All raw sequencing data were deposited to the NCBI resource under the accession number PRJNA766153.

## Supplementary Materials

The following supporting information can be downloaded at: https://www.mdpi.com/article/10.3390/biom16101464/s1, Table S1: Sequences of primers used in this study; The original western blot images can be found in Supplementary materials.
